# PD-L1-Mediated Immunosuppression in Hepatocellular Carcinoma: Relationship with Macrophages Infiltration and Inflammatory Response Activity

**DOI:** 10.3390/biom12091226

**Published:** 2022-09-02

**Authors:** Shuang Guo, Xinyue Wang, Hanxiao Zhou, Yue Gao, Peng Wang, Hui Zhi, Yue Sun, Yakun Zhang, Jing Gan, Yun Xiao, Shangwei Ning

**Affiliations:** College of Bioinformatics Science and Technology, Harbin Medical University, Harbin 150081, China

**Keywords:** PD-L1, tumor-associated macrophages, immunosuppressive tumor microenvironment, inflammatory response, immunotherapy

## Abstract

Immune dysfunction and pro-oncogenic inflammation play critical roles in malignant progression and non-response to immunotherapy for hepatocellular carcinoma (HCC). In particular, PD-1/PD-L1 blockade therapy could induce durable tumor remissions and improve the prognosis of patients to a certain extent. However, PD-L1, as a promising biomarker, has limited knowledge about its relevance to tumor microenvironment (TME) characterization and endogenous inflammatory immune responses. In this study, we systematically investigated and characterized the important intercommunication of PD-L1 with immunosuppressive TME and inflammatory response activity in HCC and predicted promising therapeutic drugs to improve the current therapeutic strategy for specific patients. We identified aberrant expression patterns of PD-L1 in HCC and completely different clinical and molecular characteristics among the PD-L1 subgroups. PD-L1 positively associated with immunosuppressive macrophages and macrophage-derived cytokines, which may contribute to the polarization of macrophages. Moreover, inflammatory response activity exhibited significant differences between high and low PD-L1 expression groups and had robust positive correlativity of the infiltration level of tumor-associated macrophages. Notably, given the immunosuppressive and inflammatory microenvironment in HCC, we screened four candidate drugs, including dasatinib, vemurafenib, topotecan and AZD6482, and corroborated in two pharmacogenomics databases, which might have potential therapeutic implications in specific HCC patients. Our results enhanced the understanding of linkage in PD-L1 expression patterns with macrophages and inflammation, which may provide new insight into the pathogenic mechanisms and potential therapeutic strategy for HCC.

## 1. Introduction

Hepatocellular carcinoma (HCC), a global health challenge, is the second most-prevalent cause of cancer-associated mortalities worldwide [1]. The incidence of HCC is growing worldwide, and by 2025, there will be an estimated incidence of >1 million individuals with liver cancer annually [2]. Despite breakthroughs in imaging technology, chemotherapy, interventional radiology, hepatic resection, and liver transplantation for early-stage HCC patients in recent years, there is no effective treatment method to date for advanced patients with dismal prognosis [3,4]. Therefore, further efforts are still demanded to develop effective therapeutic targets and alternative treatment strategies for HCC.

It is worth noting that the immune system plays a multifaceted role in HCC development, either containing tumor onset and growth through immunologic surveillance or accelerating progression through pro-tumorigenic inflammation. In particular, increased infiltration of tumor-associated macrophages (TAMs) has been implicated in high TNM stage, large tumor size, and immune suppression, ultimately resulting in tumor progression and drug resistance [5,6,7]. Meanwhile, HCC represents a classic paradigm of inflammation-related cancer, where persistent inflammation strongly influences tumor progression and therapeutic effect [8,9]. TAMs are important mediators of the link between inflammation and cancer, especially in HCC [10,11]. Notably, macrophages are heterogeneous cell populations that have been traditionally classified into two phenotypes (M1 and M2). The macrophages M1, which are classically activated, kill tumor cells and produce large amounts of pro-inflammatory cytokines. The macrophages M2, which are alternatively activated, suppress the inflammatory response as well as promote cancer development. It is important that macrophages are also plastic cells whose phenotype of polarized M1–M2 could be reversed in vitro and in vivo under certain circumstances, evading immune surveillance.

Cancer immunotherapy, including immune checkpoint inhibitors (ICIs), chimeric antigen receptor T cells, and oncolytic virotherapy, have proven their efficacy and revolutionized cancer treatment in basic and clinical studies over the past decade [12]. Especially programmed cell death protein 1 (PD-1) and programmed cell death protein ligand 1 (PD-L1), ICIs expressed on the cell surface, can activate negative regulatory and dampen antitumor immune responses, leading to the escape of cancer cells from the host immune system [13,14]. Accumulating data have suggested that therapeutic monoclonal antibodies targeting PD-1/PD-L1 have shown remarkable benefits in prolonging survival in melanoma [15], breast cancer [16], and lung cancer [17]. However, it appears that only a small percentage of HCC patients benefit from immunotherapy [18,19]. PD-L1 is up-regulated in the context of the chronic inflammation [20] and is often induced or maintained by inflammatory cytokines [14,21], which could be considered as a reflection of endogenous inflammatory immune responses. Meanwhile, the inflammatory TME plays a critical role in resistance to traditional antitumor therapies [22,23], where TAMs have been demonstrated to induce immunosuppression and diminish the efficacy of the drug sorafenib in HCC [24,25]. Nevertheless, the association between PD-L1, TAMs, and inflammatory response in HCC remains currently unclear.

Given the dissatisfied efficacy that PD-1/PD-L1-targeted cancer immunotherapy in HCC necessitates, we need to improve our understanding of the impact of PD-L1 on immune cells and inflammation. In the present study, we systematically investigated the PD-L1-related transcriptome profile of HCC and characterized its potential role of PD-L1 in the immunosuppressive TME, focusing on its relationship with TAMs and inflammatory response activities. In addition, we also identified the most promising therapeutic drugs for specialized populations among HCC patients, offering the potential to improve current therapeutic strategies.

## 2. Methods and Materials

### 2.1. Acquisition of Data

HCC gene expression profile and corresponding clinicopathological characteristics were downloaded from the Cancer Genome Atlas (TCGA, https://portal.gdc.cancer.gov/, accessed on 1 March 2021) and the Gene Expression Omnibus (GEO, https://www.ncbi.nlm.nih.gov/geo/, accessed on 1 November 2021). In the TCGA, the RNA sequencing data of 342 HCC samples and 48 normal samples were collected (TCGA LIHC cohort). Furthermore, we organized clinicopathological information on age, gender, tumor-node-metastasis (TNM), pathologic stage, World Health Organization grade, survival time and status. In addition, 81 HCC patient samples, as the validation cohort, were extracted based on the disease state in the GSE62232 cohort from GEO [26].

### 2.2. Molecular Characteristics of Different PD-L1 Subgroups

In order to investigate the Molecular characteristics of PD-L1 in HCC, the abnormally expressed genes and potential biological processes or pathways between ${\mathrm{PD}\text{-}L1}^{\mathrm{High}}$ and ${\mathrm{PD}\text{-}L1}^{\mathrm{Low}}$ subgroups were identified. The abnormally expressed genes in ${\mathrm{PD}\text{-}L1}^{\mathrm{High}}$ and ${\mathrm{PD}\text{-}L1}^{\mathrm{Low}}$ subgroups were calculated with the edgeR R package and defined by the threshold criteria of |log2 (Fold Change)| > 1 and adj. *p* value (False Discovery Rate) < 0.05 as the differentially expressed genes (DEGs). To acquire overrepresented enrichment of biological processes and pathways, we performed Gene ontology (GO) function enrichment analysis and Kyoto Encyclopedia of Genes and Genomes (KEGG) pathway enrichment analysis of the DEGs in ${\mathrm{PD}\text{-}L1}^{\mathrm{High}}$ and ${\mathrm{PD}\text{-}L1}^{\mathrm{Low}}$ subgroups using the clusterProfiler R package and followed by adj. *p* value (Benjamini-Hochbergs) < 0.05. In particular, biological processes are grouped according to functional themes by measuring semantic similarity among GO terms (GOSemSim R package) [27]. Fisher-exact tests were used to determine any differences in clinicopathological characteristics between ${\mathrm{PD}\text{-}L1}^{\mathrm{High}}$ and ${\mathrm{PD}\text{-}L1}^{\mathrm{Low}}$ subgroups.

### 2.3. The Infiltration Characteristics of Immune Cell Subpopulations

The infiltration levels of immune cell subpopulations in ${\mathrm{PD}\text{-}L1}^{\mathrm{High}}$ and ${\mathrm{PD}\text{-}L1}^{\mathrm{Low}}$ subgroups were quantified. The specific gene sets for immune cell subpopulations were obtained from previous studies by Bindea et al. [28] and Newman et al. [29]. Twenty-six types of immune cells with corresponding gene signatures were utilized for analysis, such as following activated dendritic cells (aDC), B cells, activated CD8+ T cells (CD8 T cells), cytotoxic cells, dendritic cells (DC), eosinophils, immature dendritic cells (iDC), macrophages M0, macrophages M1, macrophages M2, mast cells, neutrophils, CD56bright natural killer cells (NK CD56bright cells), CD56dim natural killer cells (NK CD56dim cells), natural killer cells (NK cells), plasmacytoid dendritic cells (pDC), T cells, T helper cells, T central memory (Tcm), T effector memory (Tem), T follicular helper cells (TFH), T gamma delta (Tgd), type-1 T helper cells (Th1 cells), type-17 T helper cells (Th17 cells), type-2 T helper cells (Th2 cells), regulatory T cells (Treg). The single sample Gene Set Enrichment Analysis (ssGSEA) was performed to quantify the immune cell infiltration levels of a single sample.

### 2.4. Inflammatory Response Activity of Different PD-L1 Subgroups

We curated gene sets for various inflammatory response biological processes using the Molecular Signatures Database (MSigDB, http://www.gsea-msigdb.org/gsea/msigdb/, accessed on 1 March 2021) C5 ontology gene sets (biological process) [30]. GSVA is commonly employed for estimating the variation in biological process and pathway activity in the samples of an expression dataset [31]. For each sample, a score for the enrichment of each inflammatory response category was calculated with GSVA analysis. The Wilcoxon test was performed to identify differences in the enrichment of inflammatory response categories between the ${\mathrm{PD}\text{-}L1}^{\mathrm{High}}$ and ${\mathrm{PD}\text{-}L1}^{\mathrm{Low}}$ subgroups.

### 2.5. Correlation of PD-L1 with Immune Cells and Cytokine Markers

We explored the correlation between the expression of PD-L1 expression, the infiltration levels of immune cells, the activation of inflammatory response and the expression of cytokine markers. The cytokine markers included the classical cytokines of macrophages M1 (IL12A, IL12B, IL23A, IL23R, IFNG and TNF) and macrophages M2 (IL4, IL10, IL13, TGFB1, TGFB2 and TGFB3) [32,33]. The correlation was calculated by Pearson correlation coefficient and defined |correlation (cor)| > 0.4 and *p* value < 0.05 is statistically significant correlation. We also utilized the Search Tool for the Retrieval of Interacting Genes Database (STRING, https://www.string-db.org/, accessed on 1 August 2021) [34] to evaluate the interaction between PD-L1 and cytokine markers.

### 2.6. Prediction of Sensitivity to Immunotherapeutic Response

The Tumor Immune Dysfunction and Exclusion (TIDE, http://tide.dfci.harvard.edu/, accessed on 1 December 2021), a computational framework, was used to evaluate the potential of tumor immune escape from the gene expression profiles of cancer samples [35,36]. We performed the TIDE algorithm to predict the TIDE score for each tumor sample to assess the response to immune checkpoint blockade (Wilcoxon test, *p* value < 0.05).

### 2.7. Estimation of Drug Response in Special Populations of HCC

Two pharmacogenomics datasets, the Cancer Therapeutics Response Portal (CTRP, https://portals.broadinstitute.org/ctrp/, accessed on 1 January 2022) [37,38] and the PRISM Repurposing dataset (PRISM, https://depmap.org/portal/prism/, accessed on 1 January 2022), provide molecular data across hundreds of cancer cell lines and large-scale drug screening, which make it possible to accurately predict drug response in samples. The human cancer cell lines (CCLs) used for subsequent CTRP and PRISM analysis were obtained from the Cancer Cell Line Encyclopedia (CCLE, https://portals.broadinstitute.org/ccle/, accessed on 1 January 2022) project [39]. The area-under-curve (AUC) values for the dose response are provided from two pharmacogenomics datasets as a measure of drug sensitivity, in which lower values indicate increased sensitivity to the treatment. In addition, messages were filtered due to the inclusion criteria: (i) cell lines with more than 20% of missing value in hematopoietic and lymphoid tissues were excluded; (ii) compounds with more than 20% of missing AUC values were excluded and other missing AUC values are imputed by applying K-Nearest Neighbor imputation; (iii) compounds common to CTRP and PRISM datasets were included. Subsequently, we calculated the AUC values of drug response using the ridge regression model (pRRophetic R package) [40] to estimate the response to candidate drugs for clinical patients in this study, and used 10-fold cross-validation based on the expression profiles and drug response data of CCLs training set to evaluate predictive accuracy (Wilcoxon test, *p* value < 0.05) [41].

## 3. Results

### 3.1. Abnormal Pattern of PD-L1 in HCC

**Figure 1:**
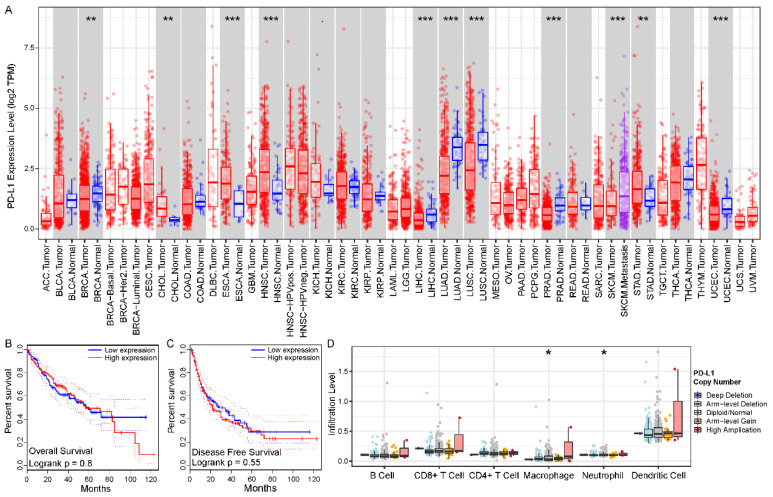
**Abnormal patterns in expression, prognosis, and immune of PD-L1 in HCC.** (**A**) Different expression levels of PD-L1 in various cancer types using TIMER. (**B**,**C**) The Kaplan-Meier survival curves showed the prognosis value of PD-L1 in overall survival and disease-free survival using GEPIA, respectively. (**D**) The differential infiltration levels of immune cells in different PD-L1 gene copy number variant types using TIMER. The asterisk character represent the statistical significance of difference, * *p* < 0.05; ** *p* < 0.01; *** *p* < 0.001.

To initially explore the role of PD-L1 in cancer, we used TIMER [42] to obtain an understanding of the expression levels of PD-L1 in each cancer type. We recognized that PD-L1 mRNA expression levels were significantly abnormal in many cancer types, in particular, its expression was significantly downregulated in HCC compared to normal tissue (*p* value < 0.001) (Figure 1A). We further explored the impact of PD-L1 expression on prognosis using GEPIA [43]. Although we did not observe a significant association of PD-L1 with overall survival or disease-free survival in the TCGA LIHC cohort (Figure 1B,C), a series of studies indicated that PD-L1 expression affects the prognosis of HCC. Next, we preliminarily investigated the impact of PD-L1 on the immune systems. We found that the immune cell infiltration levels changed with PD-L1 gene copy number, especially in macrophages and neutrophils, which significantly correlated with copy number variant type (Figure 1D).

### 3.2. The PD-L1 Subgroups Reflected Different Clinical and Molecular Characteristics

**Figure 2:**
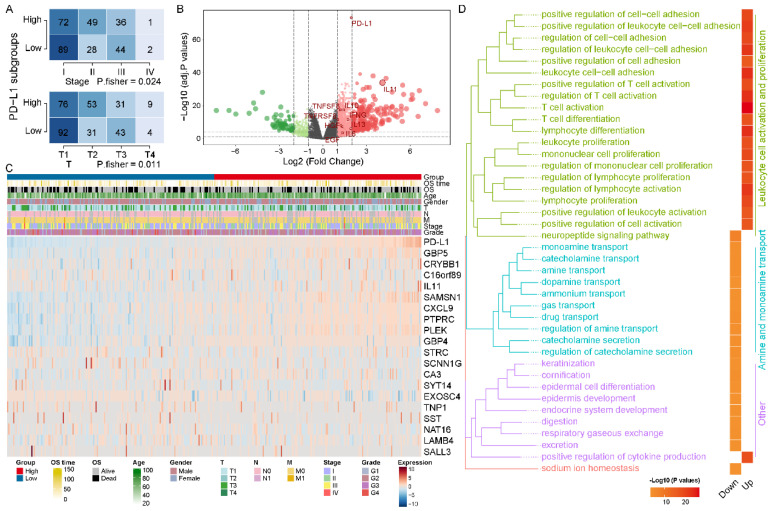
**Different molecular characteristics between**${\mathrm{PD}\text{-}L1}^{\mathrm{High}}$**and**${\mathrm{PD}\text{-}L1}^{\mathrm{Low}}$  **subgroups.** (**A**) The distribution of pathological stage and T among PD-L1 subgroups was significantly different. (**B**) The volcano plot demonstrated differences in gene expression levels, particularly for cytokines. (**C**) The heatmap charted the expression levels of TOP10 DEGs in the ${\mathrm{PD}\text{-}L1}^{\mathrm{High}}$ and ${\mathrm{PD}\text{-}L1}^{\mathrm{Low}}$ subgroups, respectively. (**D**) TOP 20 biological processes in GO summary of DEGs in PD-L1 subgroups. GO biological processes are grouped according to the functional theme and plotted for PD-L1 subgroups.

To explore the abnormal clinical and molecular characteristics among different expressions of PD-L1, we divided the HCC patients into ${\mathrm{PD}\text{-}L1}^{\mathrm{High}}$ and ${\mathrm{PD}\text{-}L1}^{\mathrm{Low}}$ subgroups by median value of PD-L1 expression. In clinicopathological characteristics, we identified pathologic stage and T distribution with significant differences between ${\mathrm{PD}\text{-}L1}^{\mathrm{High}}$ and ${\mathrm{PD}\text{-}L1}^{\mathrm{Low}}$ subgroups (Figure 2A). The results of differential expression analysis showed that 1314 genes were significantly upregulated in ${\mathrm{PD}\text{-}L1}^{\mathrm{High}}$ subgroup and 332 genes were significantly upregulated in ${\mathrm{PD}\text{-}L1}^{\mathrm{Low}}$ subgroup (Figure 2B). Figure 2C illustrated the expression levels of the top 10 DEGs in ${\mathrm{PD}\text{-}L1}^{\mathrm{High}}$ and ${\mathrm{PD}\text{-}L1}^{\mathrm{Low}}$ subgroups, respectively. Notably, we found that TAMs characteristic cytokines (such as IL10, IL13, IFNG, EGF and HGF) were significantly differentially expressed. In addition, GO enrichment analysis showed that DEGs in ${\mathrm{PD}\text{-}L1}^{\mathrm{High}}$ subgroup were significantly enriched in the biological processes of leukocyte cell activation and proliferation (such as T cell activation, regulation of T cell activation and regulation of lymphocyte activation), while DEGs in ${\mathrm{PD}\text{-}L1}^{\mathrm{Low}}$ subgroup were significantly enriched in the biological processes of amine and monoamine transport (such as regulation of amine transport and catecholamine transport) and others (Figure 2D). In parallel, KEGG analysis showed similar results; DEGs in ${\mathrm{PD}\text{-}L1}^{\mathrm{High}}$ subgroup were significantly enriched in immune-related pathways, particularly cytokines and signaling related pathways, while DEGs in ${\mathrm{PD}\text{-}L1}^{\mathrm{Low}}$ subgroup were significantly enriched in neuroactive ligand–receptor interaction pathways (Table S1).

### 3.3. PD-L1 Was Positively Correlated with Immunosuppressive Macrophages

**Figure 3:**
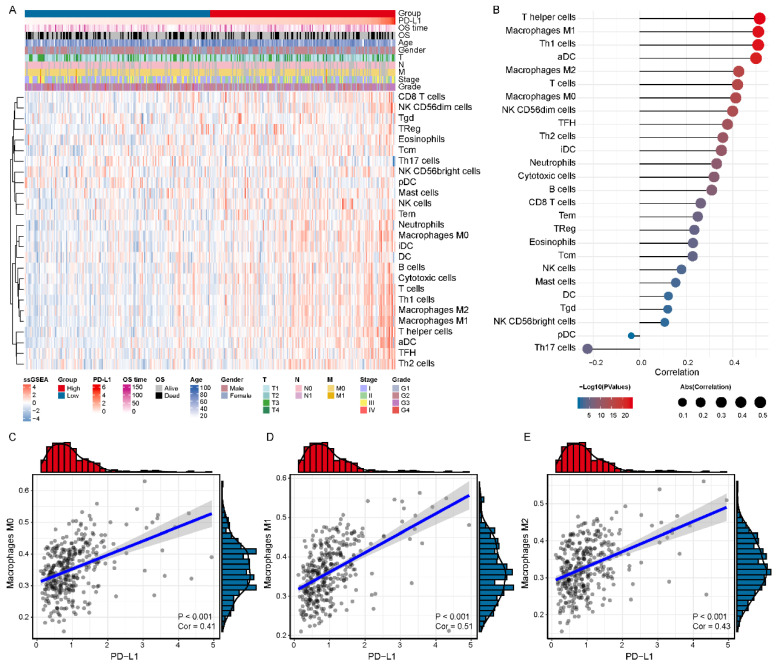
**Association of the expression level of PD-L1 with the infiltration level of immune cell subpopulations in the TCGA LIHC cohort.** (**A**) The immune landscape of HCC. (**B**) The relevance of the expression level of PD-L1 and infiltration level of immune cells. (**C**–**E**) The significant positive correlation of PD-L1 and tumor-associated macrophages (M0, M1 and M2), respectively.

To investigate the immune cell subpopulations that may correlate with PD-L1, We first assessed the infiltration level of each immune cell in the HCC microenvironment by using ssGSEA analysis (Figure 3A). Then, we calculated the association between the expression level of PD-L1 and the infiltration level of immune cells in HCC and identified seven PD-L1-associated immune cell subpopulations in the TCGA LIHC cohort (*p* value < 0.05 and cor > 0.4) (Figure 3B and Table S2). In particular, PD-L1 expression was positively correlated with the infiltration level of macrophages M0 (cor = 0.413, *p* value < 0.001), macrophages M1 (cor = 0.509, *p* value < 0.001) and M2 (cor = 0.426, *p* value < 0.001) (Figure 3C–E). In addition, all the above results were verified in the GSE62232 validation cohort (Figure S1A,B), especially with macrophages M1 (cor = 0.718, *p* value < 0.001) and M2 (cor = 0.601, *p* value < 0.001) (Figure S1C–E and Table S2). The above results indicated that PD-L1 was positively correlated with the infiltration of macrophages in HCC, which may be an important source of enhanced immunosuppression.

### 3.4. PD-L1 Was Associated with Macrophage-Derived Cytokines

**Figure 4:**
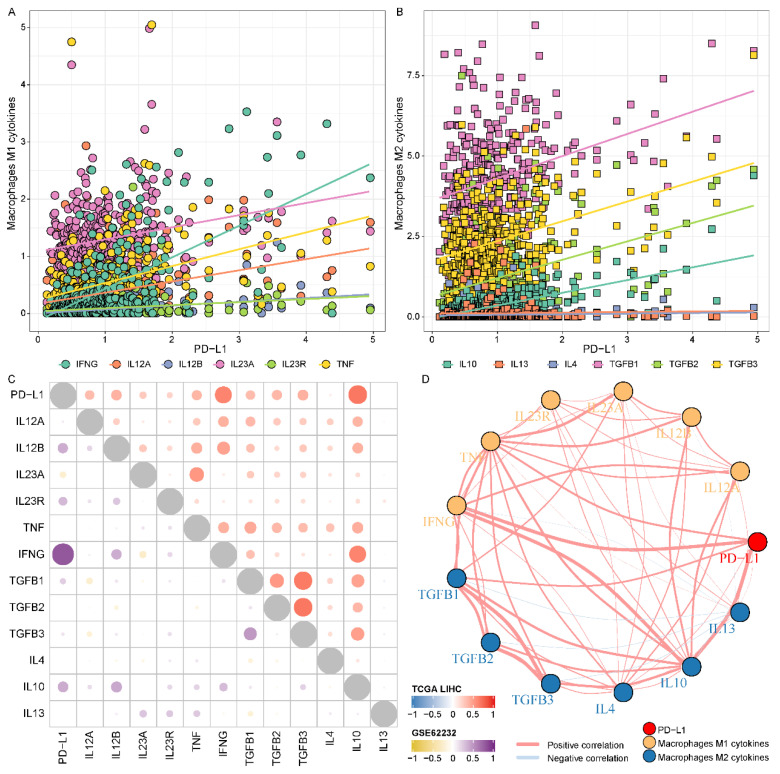
**Association of the expression level of PD-L1 and macrophage-derived cytokines in HCC.** (**A**,**B**) The relevance of the expression level of PD-L1 and macrophages (M1 and M2) related cytokines, respectively. (**C**) The correlation of PD-L1, macrophages M1 and M2 cytokines in the TCGA LIHC and GSE62232 validation cohorts. (**D**) The protein-protein interaction network of PD-L1 and cytokines.

To further elaborate on the association between PD-L1 and macrophages, we further analyzed the correlation of PD-L1 with macrophage markers, especially inflammatory response-related macrophage cytokines. The results revealed that macrophages M1-related cytokines (IL12A, IL-12B, IL23A, IL23R, TNF, and IFNG) (Figure 4A and Table S3) and macrophages M2-related cytokines (TGFB1, TGFB2, TGFB3 and IL10) (Figure 4B and Table S3) were positively correlated with PD-L1 in the TCGA LIHC cohort (*p* value < 0.001). Notably, macrophages M1 cytokine IFNG displayed a robust positive correlation with PD-L1 (cor = 0.617, *p* value < 0.001), and macrophages M2 cytokine IL10 displayed a robust positive correlation with PD-L1 (cor = 0.677, *p* value < 0.001). We also observed that most of the PD-L1 macrophages M1 and M2 cytokines showed a positive correlation with each other (Figure 4C). The analysis of the GSE62232 validation cohort also presented similar results (Figure 4C and Table S3). We also constructed a protein–protein interaction network of PD-L1 and cytokines using STRING, and multiple potential interactions were displayed between the PD-L1 and cytokines (Figure 4D and Table S4). These findings supported that PD-L1 was intimately and positively correlated with macrophages M1 and M2 and the cytokines enhanced the polarization of TAMs in an HCC microenvironment.

### 3.5. Intense Relevance to the PD-L1 Subgroups and Inflammatory Response Activity

**Figure 5:**
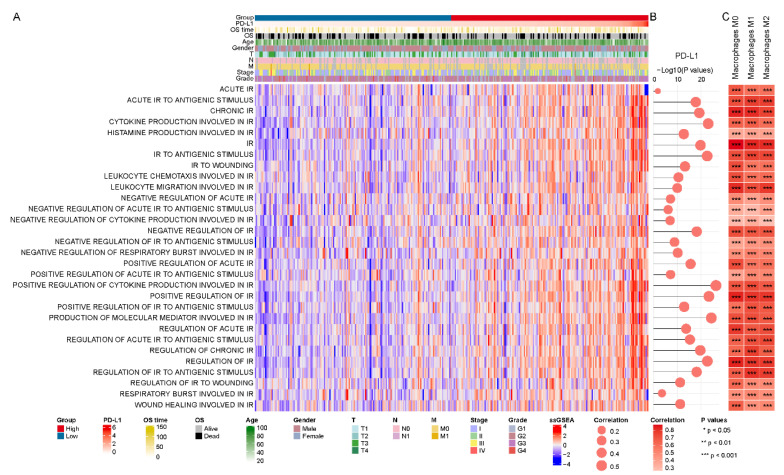
**PD-L1-related inflammatory response activity in the TCGA LIHC cohort.** (**A**) The enrichment score of inflammatory response (IR) biological processes using GSVA. (**B**,**C**) The correlation of activation of inflammatory response (IR) with the expression level of PD-L1 and infiltration level of macrophages, respectively.

DEGs in ${\mathrm{PD}\text{-}L1}^{\mathrm{High}}$ subgroup are similarly significantly enriched for multiple inflammatory response processes (Table S5). Furthermore, we observed that macrophages and cytokines, relevant to the inflammatory response, were both implicated in PD-L1. Therefore, we further explored the role of PD-L1 in inflammatory response activity in HCC. GSVA enrichment analysis showed the activation states of inflammatory response biological processes at distinct expression levels of PD-L1 in the TCGA LIHC cohort (Figure 5A). We identified that the activation of most inflammatory response processes significantly correlated with PD-L1 expression (Figure 5B) and distinctly varied among the PD-L1 subgroups (Figure S2A and Table S6), as confirmed in the GSE62232 validation cohort (Figure S2B and Table S6). Specifically, positive regulation of cytokine production involved in inflammatory response (cor = 0.538, *p* value < 0.001). Inflammatory response activity and macrophage infiltration level also had a robust positive correlation (Figure 5C). In summary, these findings indicated an important link between PD-L1 and the function of inflammatory immune response in HCC.

### 3.6. Differences in Sensitivity of Potential Therapeutic Drugs in PMI Subgroups

**Figure 6:**
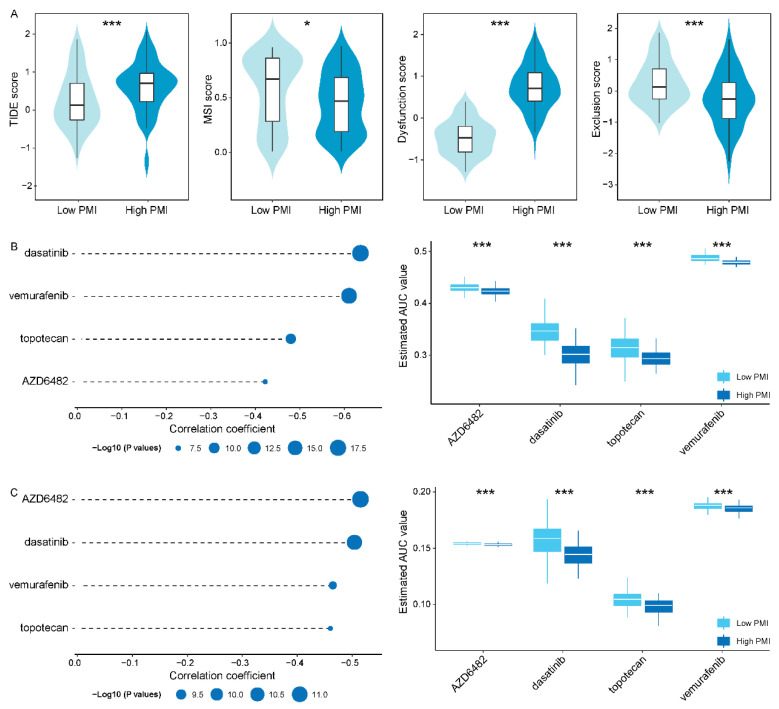
**Identification of candidate drugs with higher drug sensitivity in the**${\mathrm{PMI}}^{\mathrm{High}}$**subgroups**. (**A**) TIDE, MSI, T cell dysfunction and exclusion score in ${\mathrm{PMI}}^{\mathrm{High}}$ and ${\mathrm{PMI}}^{\mathrm{Low}}$ subgroups, respectively. (**B**,**C**) The results of Pearson correlation analysis and differential drug response analysis of four drugs in CTRP and PRISM datasets, respectively. The asterisk character represent the statistical significance of difference, * *p* < 0.05; *** *p* < 0.001.

In response to the small number of patients with durable responses to immunotherapy, we sought to identify more promising drugs for specific populations to improve treatment outcomes. We focused on a specific population with high PD-L1 expression, TAMs infiltration and high inflammatory response activity, termed ${\mathrm{PMI}}^{\mathrm{High}}$ patients, because they all have immunosuppressive mechanisms. So, we extracted and defined ${\mathrm{PMI}}^{\mathrm{High}}$ subgroup (83 patients) and ${\mathrm{PMI}}^{\mathrm{Low}}$ subgroup (84 patients), and integrated PD-L1 expression, TAMs infiltration level and inflammatory response pathway enrichment score as PMI scores. It also demonstrated that the ${\mathrm{PMI}}^{\mathrm{High}}$ subgroup displayed higher TAMs (M0, M1 and M2) infiltration level and inflammatory response activity compared to the ${\mathrm{PMI}}^{\mathrm{Low}}$ subgroup (*p* value < 0.001, Figure S3). The TIDE was utilized to evaluate the potential clinical efficacy of immunotherapy in PMI patients. We observed that the ${\mathrm{PMI}}^{\mathrm{High}}$ subgroup had a higher TIDE score and T cell dysregulation score, but a lower microsatellite instability (MSI) and T cell exclusion score (Figure 6A). In general, patients in the ${\mathrm{PMI}}^{\mathrm{High}}$ subgroup had a higher potential for tumor immune escape; hence, we urgently need to focus on their therapeutic schedule.

In order to seek candidate drugs with higher drug sensitivity in ${\mathrm{PMI}}^{\mathrm{High}}$ patients, we performed drug response prediction using CTRP- and PRISM-derived drug response data, respectively. Firstly, we screened 113 common drugs or compounds in CTRP and PRISM databases to facilitate subsequent analyses that could be corroborated with each other. Then, we performed differential drug response analysis in PMI subgroups to identify significantly different drugs or compounds, of which only lower AUC estimates in the ${\mathrm{PMI}}^{\mathrm{High}}$ subgroup were retained. Furthermore, we collected drugs or compounds with negative correlation coefficients using Pearson correlation analysis of AUC values and PMI scores (cor < −0.40). Ultimately, in the cross-corroboration of the two pharmacogenomics databases, we predicted four drugs or compounds (including dasatinib, vemurafenib, topotecan and AZD6482) with promising therapeutic potential in ${\mathrm{PMI}}^{\mathrm{High}}$ patients (Figure 6B,C and Table S7), which had lower estimated AUC values and were negatively correlated with PMI scores.

## 4. Discussion

HCC, with a 5-year survival rate of approximately 20%, has a high risk of recurrence and metastasis [44,45]. Tumor-associated macrophages are abundant in tumor and peritumoral tissues, which form immunosuppressive TME and promote chronic inflammation, and ultimately the progression of disease and resistance to conventional antitumor therapies [22,23]. Likewise, PD-L1 expression is usually induced or maintained by inflammatory cytokines [14,21,46]. However, the expression level of PD-L1 has not been used as a predictive biomarker to select patients who would benefit from treatment in clinical practice with satisfactory results [47,48,49]. Therefore, it is essential to understand the expression level and relevance of PD-L1 between pro-tumorigenic inflammation and anti-tumor immunity. In our study, we systematically characterized the abnormal pattern of PD-L1 in HCC and investigated the correlation between the expression patterns of PD-L1 and prognosis, clinicopathological features and molecular characteristics. To further understand the relationship between PD-L1 and TME as well as inflammation, we then calculated the correlation of PD-L1 expression levels with immune cells, especially TAMs and their released cytokines, and inflammatory response activity. More importantly, given that immunotherapy relying only on PD-L1 expression levels has not yielded satisfactory results, we predicted and screened potential therapeutic drugs targeting specific populations who have high PD-L1 expression levels, high macrophage infiltration levels, and high inflammatory response activities, which may provide promising therapeutic approaches for HCC PMI patients.

The overexpression of PD-L1 occurs in many common cancers and serves as a promising predictive marker for therapeutic response to PD-1/PD-L1 antibody [50]. Thus, a more comprehensive landscape of expression pattern and molecular function of PD-L1 in HCC is needed. In this study, we found that the expression pattern of PD-L1 exhibited a pathological stage- and T stage-dependent manner. In particular, stage II was accompanied by higher levels of PD-L1 expression, which may be induced by cytokines and reflect the contribution of an endogenous anti-tumor immune response, as it generally occurs in the early stages of carcinoma progression [51,52]. Unfortunately, PD-L1 was not available as a predictor of stable prognosis in our analysis, but patients with positive PD-L1 expression had shorter overall survival and disease-free survival compared to PD-L1-negative patients in several experimental studies [53,54,55,56]. The ${\mathrm{PD}\text{-}L1}^{\mathrm{High}}$ subgroup had multiple highly expressed cytokines (such as IFNG, IL10 and HGF) and its DEGs were significantly enriched in immune signaling pathways that related to leukocyte cell activation and proliferation, suggesting the essential involvement of PD-L1 in regulating TAMs function. Therefore, PD-L1 requires consideration of the additional tumor immune environment as a promising prognostic marker.

Immune cells in tumors are an important source of immunosuppression formation. Among them, TAMs, the major components of the TME, have been playing several tumor-promoting roles, such as immune suppression and neoplasm metastasis [5,6,57,58]. In response to micro-environmental stimulus, TAMs classically differentiate into macrophages M1 with pro-inflammatory and cancer-suppressive effects [59]. Alternatively, TME promotes the polarization of TAMs into macrophages M2 with anti-inflammatory and cancer-promoting properties, which produce IL-10 and suppress CD8+ T cell responses [60,61]. Previous studies have shown that high TAMs density correlates with large tumor size, high TNM stage, and metastasis [62]. In this study, we found a positive correlation between the expression level of PD-L1 and the infiltration level of TAMs (both M0, M1 and M2), which may implicate an important functional cohesion. We also noticed that PD-L1 is positively correlated with the cytokines IL10, a canonical marker of macrophages M2, which may participate in the induction and maintenance of macrophages M2-polarization and accelerate pro-tumorigenic effects in HCC.

It is well known that HCC is a classic inflammation-related cancer. Previous studies involving PD-L1 expression are usually induced or maintained by inflammatory cytokines [14,21,46]. Consequently, the up-regulation of PD-L1 expression in tumor tissues can be seen as the dominance of immunosuppression on the one hand, and response to endogenous inflammatory immune response on the other hand. In this study, we identified a strong association between PD-L1 expression and inflammatory response activity, as well as a higher inflammatory response activity accompanying the ${\mathrm{PD}\text{-}L1}^{\mathrm{High}}$ patients. Notably, the inflammatory response activity was likewise significantly and positively correlated with the infiltration level of TAMs. It is implicated that enhanced inflammation, accompanied by sustained expression of cytokines [63,64] and recruitment of immune cells to the liver [65,66,67], may promote HCC carcinogenesis and progression by inducing immunosuppression formation and accelerating cancer cell growth [66,68].

Despite immunotherapy bringing hopefulness to oncology patients, only a minority of patients currently have a durable response to immunotherapy. The efficacy of the inhibitor sorafenib is limited in HCC patients, because intrinsic therapeutic resistance contributes to the development of intolerance and drug resistance [25]. Zhou et al. demonstrated that TAMs induce immunosuppression [24], promoting tumor development and resistance to sorafenib [69]. Furthermore, an important driver of oxaliplatin resistance has been reported to be that TAMs trigger autophagy and apoptosis evasion of HCC cells [70]. To more precisely target HCC patients, we defined specific populations, called PMI subgroups, by combining the characteristics of PD-L1 expression, TAMs infiltration, and inflammatory response activity. The results showed that the ${\mathrm{PMI}}^{\mathrm{High}}$ subgroup had a higher immune evasion potentiality and lower microsatellite instability, indicating that for specific patients it would be more challenging to benefit from immunotherapy. To provide promising population-based therapeutic strategies, we predicted four reliable drugs (including dasatinib, vemurafenib, topotecan and AZD6482) for the ${\mathrm{PMI}}^{\mathrm{High}}$ patients, which were effective in the prediction model of drug response from both CTRP and PRISM datasets. Immunotherapy and chemotherapy will likely maximize the immune-stimulating effect of therapeutic drugs through individualized dosing of patients in specific populations.

## 5. Conclusions

In summary, our study provided a systematic analysis of aberrant patterns of PD-L1 in HCC to assess the association between PD-L1 expression, immunosuppressive TME (especially TAMs), macrophage-derived cytokines and inflammatory response activity. In addition, we specifically screened and primarily validated four candidate drugs (including dasatinib, vemurafenib, topotecan and AZD6482) with high confidence in both CTRP and PRISM databases for ${\mathrm{PMI}}^{\mathrm{High}}$ patients against the microenvironment of immune suppression and chronic inflammation in HCC. This comprehensive analysis has greatly improved our understanding of the relationship between PD-L1 expression, immune cells infiltration, and inflammatory response activity, which may be valuable in deciphering immune escape and neoplastic progression, and provided insights into patient stratification and therapeutic drugs to optimize precision medicine.

## Data Availability

All data used in our study are publicly available. RNA-seq data and corresponding clinicopathological characteristics of LIHC were downloaded from TCGA (https://tcga-data.nci.nih.gov/tcga/, accessed on 1 March 2021). The data of GSE62232 were obtained from GEO (https://www.ncbi.nlm.nih.gov/geo/, accessed on 1 November 2021). The human CCLs were accessed through CCLE (https://portals.broadinstitute.org/ccle/, accessed on 1 January 2022). The drug response data of CCLs were available through CTRP (https://portals.broadinstitute.org/ctrp/, accessed on 1 January 2022) and PRISM (https://depmap.org/portal/prism/, accessed on 1 January 2022) databases.

## Supplementary Materials

The following are available online at https://www.mdpi.com/article/10.3390/biom12091226/s1. Figure S1. Association of the expression level of PD-L1 with the infiltration level of immune cell subpopulations in the GSE62232 validation cohort; Figure S2. The different inflammatory responses between ${\mathrm{PD}\text{-}L1}^{\mathrm{High}}$ and ${\mathrm{PD}\text{-}L1}^{\mathrm{Low}}$ subgroups; Figure S3. Differential levels of TAMs infiltration and inflammatory response activity between ${\mathrm{PMI}}^{\mathrm{High}}$ and ${\mathrm{PMI}}^{\mathrm{Low}}$ subgroups; Table S1. The significant enrichment of the KEGG pathways in PD-L1 subgroups; Table S2. The relevance of the expression level of PD-L1 and infiltration level of immune cells in the TCGA LIHC and GSE62232 validation cohorts; Table S3. The correlation of PD-L1, macrophages M1 and M2 cytokines in the TCGA LIHC and GSE62232 validation cohorts; Table S4. The protein-protein interaction network of PD-L1 and cytokines; Table S5. The significant enrichment of inflammatory response-related biological processes in PD-L1 subgroups; Table S6. The difference in inflammatory response among PD-L1 subgroups; Table S7. The drug response prediction results of candidate drugs in CTRP and PRISM databases.

## Abbreviations

HCC: hepatocellular carcinoma; TME: tumor microenvironment; TAMs: tumor-associated macrophages; ICIs: immune checkpoint inhibitors; PD-1: programmed cell death protein 1; PD-L1: programmed cell death protein ligand 1; TCGA: the Cancer Genome Atlas; GEO: Gene Expression Omnibus; TNM: tumor-node-metastasis; DEGs: differentially expressed genes; GO: Gene ontology; KEGG: Kyoto Encyclopedia of Genes and Genomes; ssGSEA: single sample Gene Set Enrichment Analysis; MSigDB: Molecular Signatures Database; STRING: Search Tool for the Retrieval of Interacting Genes Database; TIDE: Tumor Immune Dysfunction and Exclusion; CTRP: Cancer Therapeutics Response Portal; CCLs: cancer cell lines; CCLE: Cancer Cell Line Encyclopedia; AUC: area under curve.
