# Supplementary material for: PD-L1-Mediated Immunosuppression in Hepatocellular Carcinoma: Relationship with Macrophages Infiltration and Inflammatory Response Activity
Source: Biomolecules. 2022 Sep 2;12(9):1226. doi: 10.3390/biom12091226 (PMC9496538; doi:10.3390/biom12091226)
Supplement: Supplementary file 1 [file biomolecules-12-01226-s001.zip › Supplementary Files/Supplementary Figures.pdf]

Supplementary Figures

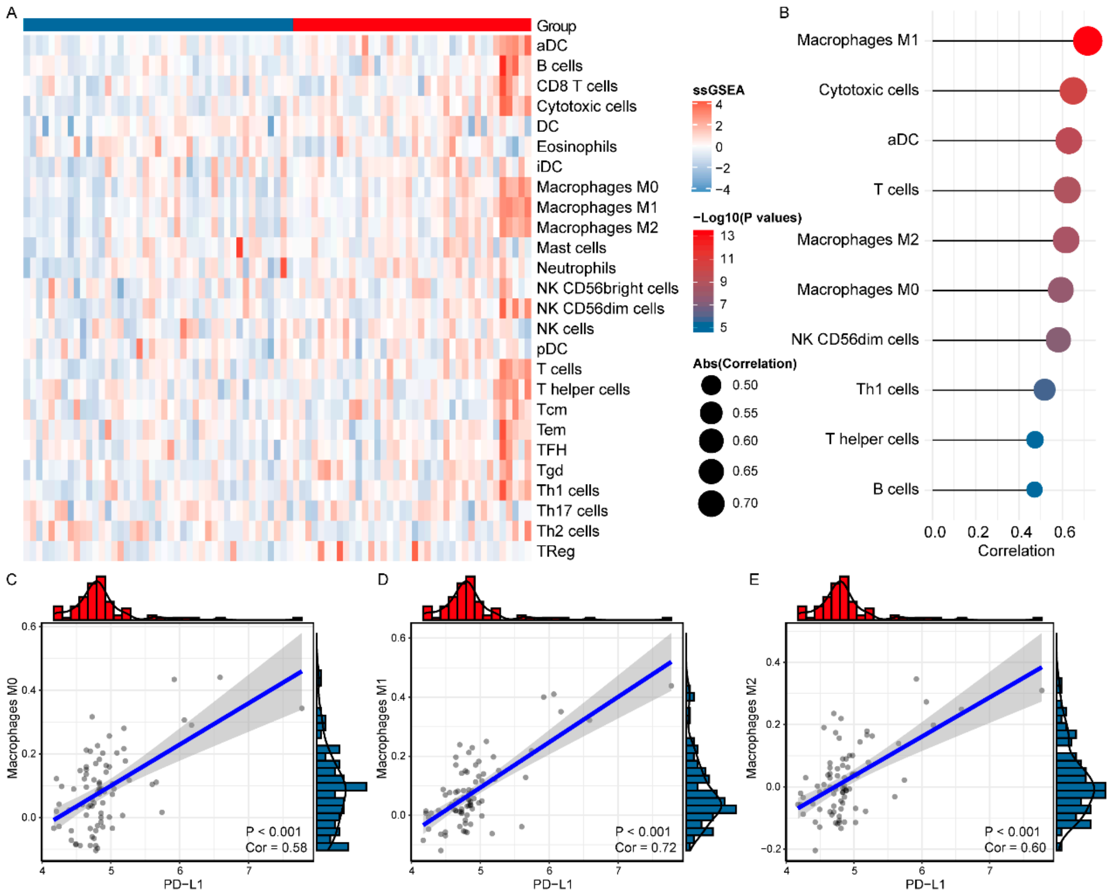

Figure S1. Association of the expression level of PD-L1 with the infiltration level of immune cell subpopulations in the GSE62232 validation cohort.

(A) The immune cell profiling of microenvironment in GSE62232. **(B)** The correlation of the expression level of PD-L1 with infiltration level of immune cells. **(C-E)** PD-L1 and macrophages (M0, M1 and M2) were associated positively, respectively.

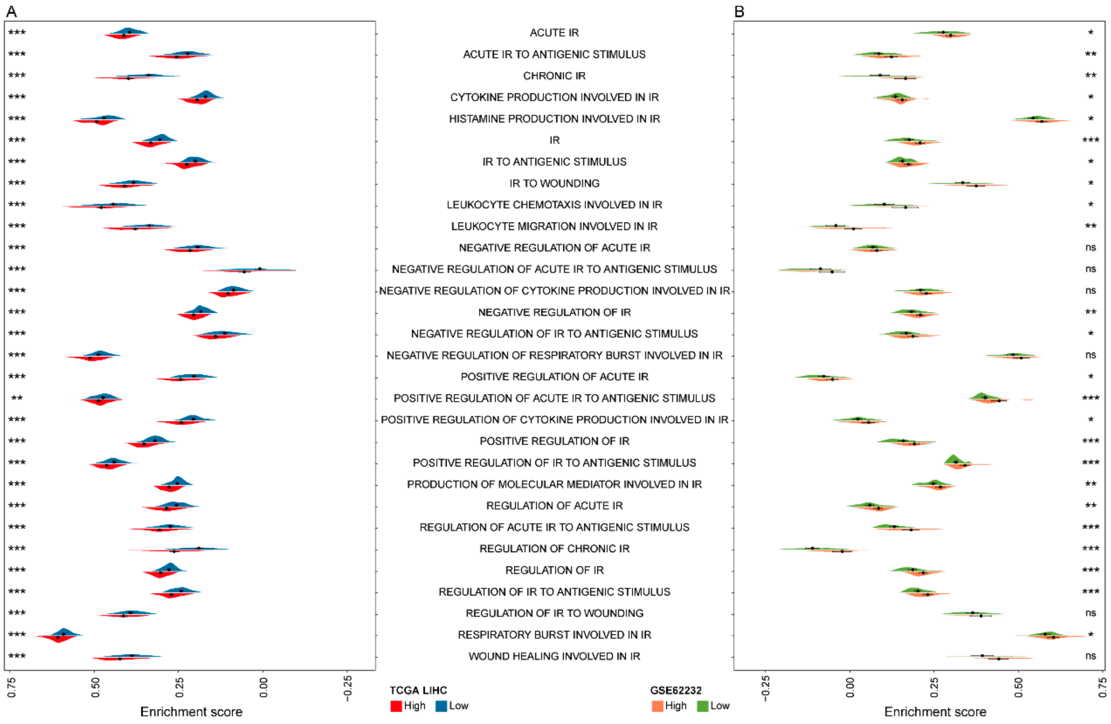

Figure S2. The different inflammatory responses between PD-L1^{High} and PD-L1^{Low} subgroups.

(A-B) The differences in the activity of inflammatory response (IR) processes between the PD-L1^{High} and PD-L1^{Low} subgroups from the TCGA LIHC and GSE62232 validation cohorts, respectively. The asterisk character represent the statistical significance of difference: ns, $p > 0.05$; *$p < 0.05$; **$p < 0.01$; ***$p < 0.001$.

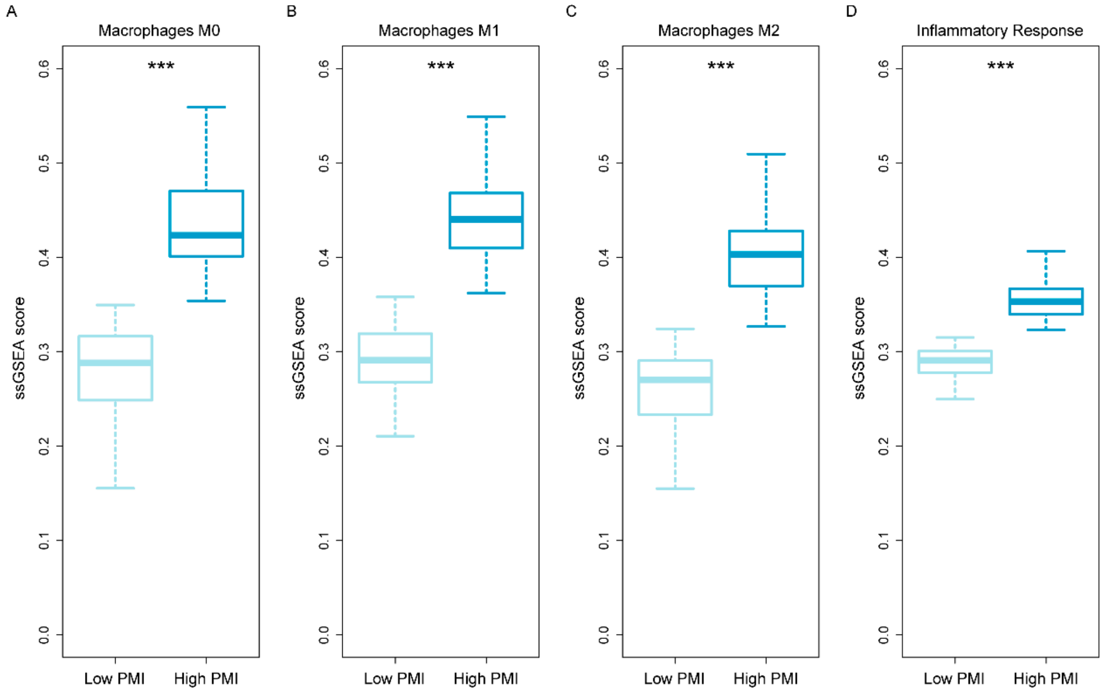

Figure S3. Differential levels of TAMs infiltration and inflammatory response

activity between PMI^{High} and PMI^{Low} subgroups.

(A-D) The differences in macrophages (M0, M1 and M2) infiltration and inflammatory response activity between the PMI^{High} and PMI^{Low} subgroups, respectively. The asterisk character represent the statistical significance of difference: ns, $p > 0.05$; *$p < 0.05$; **$p < 0.01$; ***$p < 0.001$.
